# Predicting severity of adverse cardiorespiratory effects of morphine in premature infants: a *post hoc* analysis of Procedural Pain in Premature Infants trial data

**DOI:** 10.1016/j.bja.2020.10.034

**Published:** 2020-12-09

**Authors:** Caroline Hartley, Luke Baxter, Fiona Moultrie, Ryan Purdy, Aomesh Bhatt, Richard Rogers, Chetan Patel, Eleri Adams, Rebeccah Slater

**Affiliations:** 1 Department of Paediatrics, University of Oxford, Oxford, UK; 2 Department of Anaesthetics, John Radcliffe Hospital, Oxford University Hospitals NHS Foundation Trust, Oxford, UK; 3 Department of Ophthalmology, John Radcliffe Hospital, Oxford University Hospitals NHS Foundation Trust, Oxford, UK; 4 Newborn Care Unit, John Radcliffe Hospital, Oxford University Hospitals NHS Foundation Trust, Oxford, UK

**Keywords:** adverse effects, machine learning, morphine, personalised medicine, predictive modelling, preterm infant, respiratory depression

Editor—Hospitalised infants can experience pain during essential clinical procedures. However, pharmacological analgesics are infrequently prescribed, often out of fear of adverse effects.^1^^,^^2^ Opioids are commonly administered analgesics, but are associated with a risk of adverse cardiorespiratory events, and therefore are primarily prescribed to ventilated infants.^3^ Neonatologists face considerable challenges when trying to ensure a balance between obtaining clinically significant analgesia whilst minimising adverse effects.^4^ Predicting an individual's likelihood of adverse drug effects could facilitate tailored dosing. This likelihood will be related to individual variation in pharmacokinetic factors. However, baseline physiological stability may play a role; for example, a relatively unstable infant may have lower resilience to adverse cardiorespiratory events.

We previously conducted the Procedural Pain in Premature Infants (Poppi) trial, a randomised placebo-controlled trial investigating the analgesic efficacy and safety of oral morphine in non-ventilated premature infants.^5^ The trial was stopped early because of an unacceptable risk of harm without evidence of analgesic benefit. We electronically captured vital signs for 24 h before and after the clinical procedure (a medically required heel lance and retinopathy of prematurity [ROP] screening). Although validating predictive models for clinical use requires large data sets,^6^^,^^7^ these limited but unique and comprehensive data provide a valuable opportunity to investigate the physiological factors predisposing infants to morphine-related adverse cardiorespiratory effects.

We conducted a *post hoc* analysis of Poppi trial data. Written informed parental consent and approval from the Medicines and Healthcare Products Regulatory Agency and Northampton Research Ethics Committee (15/EM/0310) were obtained. Full details of recruitment, original trial design, and procedures are given elsewhere.^5^

Fifteen infants in the trial received oral morphine (100 μg kg^−1^) ∼1 h before the clinical procedure. Although all infants were deemed clinically stable, there was wide variation in their baseline physiological stability (Supplementary Fig 1). Individual baseline physiological stability data and subject characteristics are provided in Supplementary Tables 1 and 2.

After drug administration, infants who received morphine had a significant reduction in HR compared with infants who received placebo,^5^ and the magnitude of this drop varied greatly between individuals (Supplementary Fig 2; Supplementary Table 1). Similarly, there was substantial individual variation in respiratory adverse effects (Supplementary Table 1). Using machine learning with multivariate linear regression, we investigated whether the combined risk of cardiorespiratory adverse effects (cardiorespiratory adverse effects score; see Supplementary methods) can be predicted in individual infants from their baseline physiological stability (number of episodes of profound oxygen desaturation, whether an infant experienced episodes of apnoea, average HR, and average respiratory rate) and postmenstrual age (PMA). The model strongly predicted the overall cardiorespiratory adverse effects score (Fig 1a; *R*^2^=0.57 [*P*=0.010]; median absolute error=0.97 [*P*=0.011]). Univariate linear regression confirmed that each of the baseline physiological variables correlated with the magnitude of both the cardiac and respiratory adverse effects (Supplementary Fig 3; Fig. 1b). Although PMA had minimal predictive value (Fig 1b), this may be attributable to the narrow age range of infants (34–38 weeks) that were included; previous pharmacodynamic studies have demonstrated the effect of prematurity on morphine clearance.^8^

**Fig 1:**
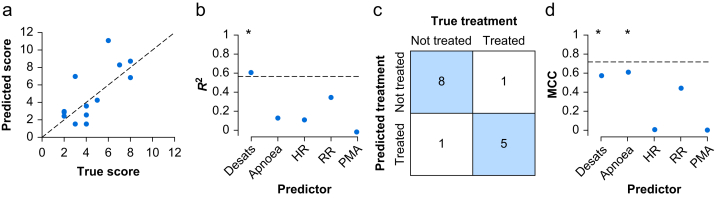
Baseline physiological stability is predictive of cardiorespiratory adverse effects. (a) The predicted cardiorespiratory adverse effects score from the model compared with the true cardiorespiratory adverse effects score for each infant. The dashed line indicates perfect prediction (*y*=*x*). (b) The *R*^2^ value for models built from each baseline predictor individually to predict the cardiorespiratory adverse effects score (Apnoea, whether an infant experienced episodes of apnoea; Desats, the number of episodes of profound oxygen desaturation; HR, average heart rate; PMA, postmenstrual age; RR, average respiratory rate). The dashed line indicates the *R*^2^ value for the full model with all five baseline predictors. ∗*P*<0.05 *R*^2^ values of the univariate model. (c) Confusion matrix comparing the number of infants who were predicted from the classification model to be treated for respiratory adverse effects, compared with their true treatment (each box indicates the number of infants). (d) Matthew's correlation coefficient (MCC) for classification models built from each baseline predictor individually to predict whether or not an infant was treated for respiratory adverse effects. Dashed line indicates the MCC value for the full model with all five baseline predictors. ∗*P*<0.05 MCC values in the univariate model.

Six infants who received morphine developed significant adverse effects that required treatment with resuscitative noninvasive positive-pressure ventilation or increased respiratory support. Using the same five baseline variables in a classification model, we could predict whether or not infants required treatment for respiratory adverse effects with an accuracy of 87% (*P*=0.009; Matthew's correlation coefficient=0.72 [*P*=0.012]; Fig 1c). The multivariate model performed better than any univariate predictor model (Fig 1d). Using this model, we would predict that if the 15 placebo-treated infants had received morphine, then 10 of them would have required treatment for respiratory adverse effects.

Lastly, individual pharmacokinetic variation will affect both adverse and therapeutic effects of the drug. We investigated whether infants who required treatment for respiratory adverse effects had lower pain-related outcomes. The magnitude of the noxious-evoked brain activity after heel lancing was significantly lower in infants who received treatment for respiratory adverse effects than in infants who did not receive treatment (Supplementary Fig 4a; mean difference=–0.97; *P*=0.005). However, there was no significant difference between the two groups in the Premature Infant Pain Profile-Revised score (a composite behavioural and physiological pain score) after ROP screening (Supplementary Fig 4b; mean difference=1.83; *P*=0.84) or heel lancing (Supplementary Fig 4c; mean difference=–0.89; *P*=0.32), which may be attributable to a lack of power in this small sample or relate to the limitations of behavioural and physiological measures in discriminating pain from distress.^9^ Although the small sample means caution is needed when interpreting this result, the apparent relationship between morphine-related adverse effects and noxious-evoked brain activity suggests the lack of a therapeutic window for oral morphine in non-ventilated infants. However, further trials are warranted in ventilated infants, where respiratory adverse consequences can be mitigated.

We show that infant baseline physiological stability is predictive of adverse cardiorespiratory effects, and independently that infants experiencing the greatest adverse effects have significantly reduced noxious-evoked brain activity. Nevertheless, this should not be interpreted to suggest that infants who are more physiologically unstable before morphine administration will have lower noxious-evoked brain activity (i.e. a correlation between A and B, and between B and C does not prove a link from A to C). These relationships are mechanistically distinct.

In summary, the potential adverse effects for all drugs must be carefully weighed against benefits and the acceptable balance is always context dependent.^4^ The Poppi trial was stopped early, so this analysis was limited to 15 infants. Given the sample size, the models should be validated independently before being used in a clinical context.^6^ However, this analysis shows the potential of using modelling to predict which infants are at risk of adverse effects from analgesics and further highlights the value of physiological monitoring to optimise pharmacotherapy in individual infants.^10^ Application of this modelling approach could facilitate personalised drug dosing, which takes into account the individual infant; the targeted provision of appropriate monitoring; or pre-emptive optimisation of respiratory support, ultimately safeguarding infants against iatrogenic harm.

## Declarations of interest

AB has shares and investment trusts held indirectly and through pension funds in the following publicly listed pharmaceutical companies: GSK, Hikma, ObsEva, and RB. All other authors have no competing interests.

## References

[1] Hartling L., Ali S., Dryden D.M. (2016). How safe are common analgesics for the treatment of acute pain for children? A systematic review. Pain Res Manag.

[2] Roofthooft D.W.E., Simons S.H.P., Anand K.J.S., Tibboel D., van Dijk M. (2014). Eight years later, are we still hurting newborn infants?. Neonatology.

[3] Carbajal R., Eriksson M., Courtois E. (2015). Sedation and analgesia practices in neonatal intensive care units (EUROPAIN): results from a prospective cohort study. Lancet Respir Med.

[4] Moultrie F., Shriver A., Hartley C. (2019). A universal right to pain relief: balancing the risks in a vulnerable patient population. Lancet Child Adolesc Health.

[5] Hartley C., Moultrie F., Hoskin A. (2018). Analgesic efficacy and safety of morphine in the Procedural Pain in Premature Infants (Poppi) study: randomised placebo-controlled trial. Lancet.

[6] Poldrack R.A., Huckins G., Varoquaux G. (2020). Establishment of best practices for evidence for prediction: a review. JAMA Psychiatry.

[7] Varoquaux G. (2018). Cross-validation failure: small sample sizes lead to large error bars. Neuroimage.

[8] Saarenmaa E., Neuvonen P.J., Rosenberg P., Fellman V. (2000). Morphine clearance and effects in newborn infants in relation to gestational age. Clin Pharmacol Ther.

[9] Slater R. (2019). The challenge of distinguishing pain from distress in young children. Lancet Child Adolesc Health.

[10] Poppe J.A., van Weteringen W., Völler S. (2020). Use of continuous physiological monitor data to evaluate doxapram therapy in preterm infants. Neonatology.

