# Supplementary material for: Predicting severity of adverse cardiorespiratory effects of morphine in premature infants: a post hoc analysis of Procedural Pain in Premature Infants trial data
Source: Br J Anaesth. 2020 Dec 9;126(4):e133–5. doi: 10.1016/j.bja.2020.10.034 (PMC8767644; doi:10.1016/j.bja.2020.10.034)
Supplement: Multimedia component 1 [file mmc1.pdf]

Supplementary Material:
Predicting the severity of adverse cardiorespiratory effects of morphine in premature infants: a post-hoc analysis of Poppi trial data

Caroline Hartley, Luke Baxter, Fiona Moultrie, Ryan Purdy, Aomesh Bhatt, Richard Rogers, Chetan Patel, Eleri Adams and Rebeccah Slater

Supplementary Methods

Full details of the recruitment, trial design and trial procedures are given elsewhere[1–3]. Briefly, infants were aged between 34-42 weeks postmenstrual age (PMA) at the time of study and had been born at less than 32 weeks' gestation or with a birthweight lower than 1501 g, fulfilling national criteria for retinopathy of prematurity (ROP) screening. Infants were given oral morphine (n=15 infants) or placebo (n=15) approximately 1 hour prior to the clinical procedure - a medically-required heel lance for blood sampling and a ROP screening. The co-primary outcome measures were the Premature Infant Pain Profile – Revised (PIPP-R) score (a composite behavioural and physiological pain score) following ROP screening and the magnitude of the noxious-evoked brain activity recorded using EEG (electroencephalography) following heel lance. Physiological variables (oxygen saturation, heart rate, respiratory rate) were recorded for 24 hours before and after the clinical procedure.

Recording techniques

Continuous heart rate derived from an ECG, respiratory rate from an impedance pneumograph, and oxygen saturation from a pulse oximeter were recorded at a sampling rate of 1 Hz using an IntelliVue MX800 Phillips monitor and downloaded continuously using ixTrend software (ixcellence GmbH, Germany). The time of drug administration, heel lancing, and ROP screening were electronically marked on the physiological recordings by a researcher in real time.

Brain activity was recorded using EEG at a sampling rate of 2 kHz, with 8 recording electrodes (including Cz) positioned on the scalp and referenced to Fz, with a ground electrode on the forehead. The heel lance was time-locked to the electrophysiological recordings using an event-detection interface[4]. A hand-held camera was used to video the infant's facial expression throughout the clinical procedure for PIPP-R scoring. The timing of the heel lance was synchronised on the video recording using an LED light, which was activated by the researcher at the moment of triggering the heel lance.

Analysis

Identifying episodes of physiological instability

As defined in the Poppi trial[1], episodes of oxygen desaturation were identified as periods during which oxygen saturations fell below 80% for at least 10 seconds. Apnoeic episodes were identified from clinical records, or by retrospective review of the impedance pneumograph for breathing pauses lasting longer than 20 seconds during bradycardic episodes.

Apnoeic episodes requiring resuscitative non-invasive positive pressure ventilation (NIPPV) and increased respiratory support were recorded by the research and clinical team. Increased respiratory support was defined as a significant increase in oxygen requirement or an increase in 'respiratory support modality'. A significant increase in oxygen requirement was defined as an increase in oxygen supply by more than 10%, a flow rate change of more than 1 litre/minute

(if receiving high flow therapy) or a flow rate change of more than 0.04 litres/minute (if receiving low flow oxygen). 'Respiratory support modality' was graded from 1–4, according to the following definitions:

Grade 1: self-ventilating on room air
Grade 2: low flow (0.01–0.35 litres/minute; 100% oxygen)
Grade 3: high flow (1–8 litres/minute) or continuous positive airway pressure (CPAP) or duoPAP (21–100% oxygen)
Grade 4: ventilator (21–100% oxygen)

Infants who required treatment for respiratory side effects were defined as those infants who had an increase in respiratory support and/or NIPPV.

Baseline physiological stability

Baseline physiological stability was evaluated from physiological variables recorded prior to morphine administration over an approximately 23-hour period (vital signs monitoring was conducted for 24 hours prior to the clinical procedure, and administration of morphine occurred approximately 1-hour prior this). The mean heart rate, mean respiratory rate, number of oxygen desaturations, and whether or not the infant experienced episodes of apnoea were calculated during this period.

Calculating the magnitude of the heart rate drop following morphine administration

The magnitude of the heart rate drop following morphine administration was calculated for each infant as the absolute value of the area-under-the-curve of the heart rate drop below one standard deviation below the mean of the baseline (prior to morphine administration, Supplementary Figure 2). In more detail, the magnitude of the heart rate drop following morphine administration was calculated for each infant from their average heart rate time course. Average heart rate time courses were calculated as the mean heart rate (for each infant) in 1-hour moving windows, with a sliding window step of 0.5 hours. Each time course was mean-centred (i.e. the mean was subtracted) to the average heart rate prior to drug administration. The start of the heart rate drop was identified as the first point at which the heart rate fell below a threshold level following morphine administration, and the end of the heart rate drop as the time at which the heart rate rose back above this threshold (or the end of the recording if the heart rate did not rise above the threshold) (Supplementary Figure 2). The threshold was set as one standard deviation (SD) below the mean of the heart rate prior to drug administration (note that the data was mean-centred, so this threshold equates to -1SD). The start of the heart rate drop had to be within 4 hours of drug administration. If the heart rate immediately rose above threshold after the start of the heart rate drop (within 1 hour, i.e. 2 time points), and then fell again below the threshold, the second point was taken as the start of the heart rate drop. Similarly, at the end of the heart rate drop, if the heart rate went above threshold but then immediately (within 1 hour, i.e. 2 time points) decreased below the threshold again this was taken as a continuous drop in heart rate and the end of the heart rate drop was taken as the time at which the heart rate rose above threshold for the second time. To exclude fluctuations in heart rate that would be expected by chance, the heart rate drop was only counted if it lasted for longer than 1 hour (i.e. 2 time points, Supplementary Figure 2D). The magnitude of the heart rate drop for each infant was calculated as the absolute value of the area-under-the-curve of the heart rate between the start and end of the heart rate drop following morphine administration, thus providing a composite measure of the duration and amplitude of the heart rate drop (Supplementary Figure 2).

Respiratory Adverse Effects Score

Infants were given a Respiratory Adverse Effects Score based on the severity of episodes of apnoea and desaturation (Supplementary Table 3).

Cardiorespiratory Adverse Effects Score

Respiratory and cardiac effects were combined into an overall Cardiorespiratory Adverse Effects Score (range 0-8). To allow equal weighting of the heart rate and respiratory effects, a Heart Rate Score (range 0-4) was given to each infant (Supplementary Table 3). The Cardiorespiratory Adverse Effects Score was calculated as the sum of the Heart Rate Score and Respiratory Adverse Effects Score.

Magnitude of noxious-evoked brain activity and PIPP-R scores

The magnitude of the noxious-evoked brain activity following the heel lance and the PIPP-R (Premature Infant Pain Profile – Revised) scores were calculated as previously described[1 5]. The magnitude of the noxious-evoked brain activity following the heel lance was calculated at the Cz electrode in the time window 400-700 ms after the stimulus using a validated EEG-based template of noxious-evoked brain activity [1 5]. EEG traces were first Woody filtered, with a maximum jitter of ± 50ms, in the region of 400—700 ms after the stimulus, by identifying the maximum correlation with the template. PIPP-R (Premature Infant Pain Profile – Revised) scores were calculated from the video recordings and physiological recordings [1]. The PIPP-R score for the heel lance was calculated in the 30-second period following the heel lance, during which time the foot was not squeezed to ensure the response was related to the noxious input from the lancet. The PIPP-R score following the ROP screening was calculated in the 30-second period after removal of the speculum from the second eye. Facial expressions were rated independently by two trained investigators.

Statistics

Model training and analyses were carried out in MATLAB (2018a, Mathworks). Multivariate linear regression was used to predict the Cardiorespiratory Adverse Effects Score from five pre-drug administration baseline predictors (PMA, number of oxygen desaturations, mean heart rate, mean respiratory rate, whether the infant had episodes of apnoea). Prediction performance was assessed using the sums-of-squares formulation of the coefficient of determination (R^2) and the median absolute error (MAE) with leave-one-out cross-validation. To test for statistical significance of the MAE and R^2 values, permutation testing was performed comparing the trained model with 5000 random permutations of the data, performed using FSLs PALM software[6]. Within each training set of the leave-one-out cross validation outliers were identified and removed (from that training set), by using linear regression with each baseline predictor independently and thresholding the Cook's distance at a value of 1. All infants are included in assessing model performance. Univariate models for each baseline predictor used linear regression with leave-one-out cross validation and outlier rejection within each training set.

We used a multivariate binomial logistic regression classification model to predict whether or not an infant required treatment for respiratory adverse effects from the five baseline variables. Model performance was assessed using accuracy and Matthew's correlation coefficient (MCC), with leave-one-out cross validation. To test for statistical significance, the model was compared with 5000 random permutations of the data. Within each training set of the leave-one-out cross validation outliers were identified and removed as above. Univariate models for each baseline predictor used binomial logistic regression classification with leave-one-out

cross validation, and outlier rejection within each training set. To apply the model to infants who received placebo, the classification model was trained on the data from all infants who received morphine using all five baseline variables and then used to predict the classification from the baseline variables of the placebo-treated infants.

Finally, a one-tailed non-parametric t-test was used to determine whether the magnitude of noxious-evoked brain activity and PIPP-R scores were lower in infants that had treatment for respiratory adverse effects compared with infants who did not have treatment. Statistical significance was assessed using permutation testing (5000 random permutations).

Supplementary Figures

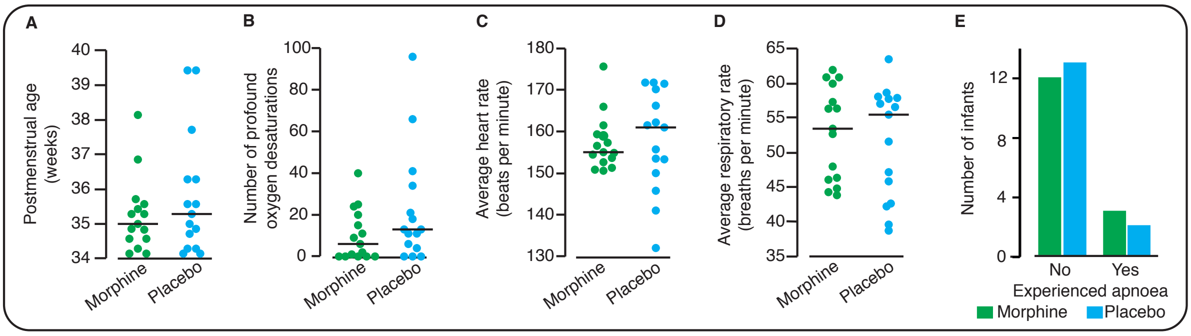

Supplementary Figure 1: Baseline physiological variables and PMA. All infants were considered stable by the treating physician but large variability in physiological stability prior to drug administration was apparent. Each dot indicates an individual infant. Green – infants who received morphine, blue – infants who received placebo. Black horizontal lines indicate the median. (E) Histogram indicating the number of infants who experienced episodes of apnoea in the baseline period. All measures are calculated in the 23 hours prior to drug administration.

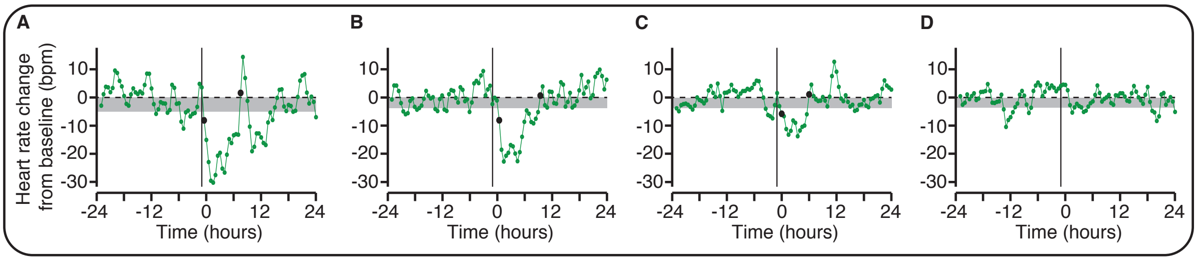

Supplementary Figure 2: Calculating the magnitude of the heart rate change. The average heart rate (calculated in 1-hour intervals with a sliding window of 30 minutes) across the 48-hour recording period in 4 infants, baseline corrected to the average pre-drug administration. The start of the heart rate drop (first black dot) is the first time point at which the heart rate drops below a threshold of one standard deviation of the baseline (grey shaded region). The end of the heart rate drop was the point at which the heart rate rose above this threshold (see Supplementary Methods). Time = 0 is the time of the clinical procedure. The black vertical line indicates morphine administration. (D) This infant was the only one (of the 15) who did not have a heart rate drop following morphine administration. Although the heart rate falls below threshold, this is only for one time point and so is excluded to account for random fluctuations (which can also be observed in the baseline period).

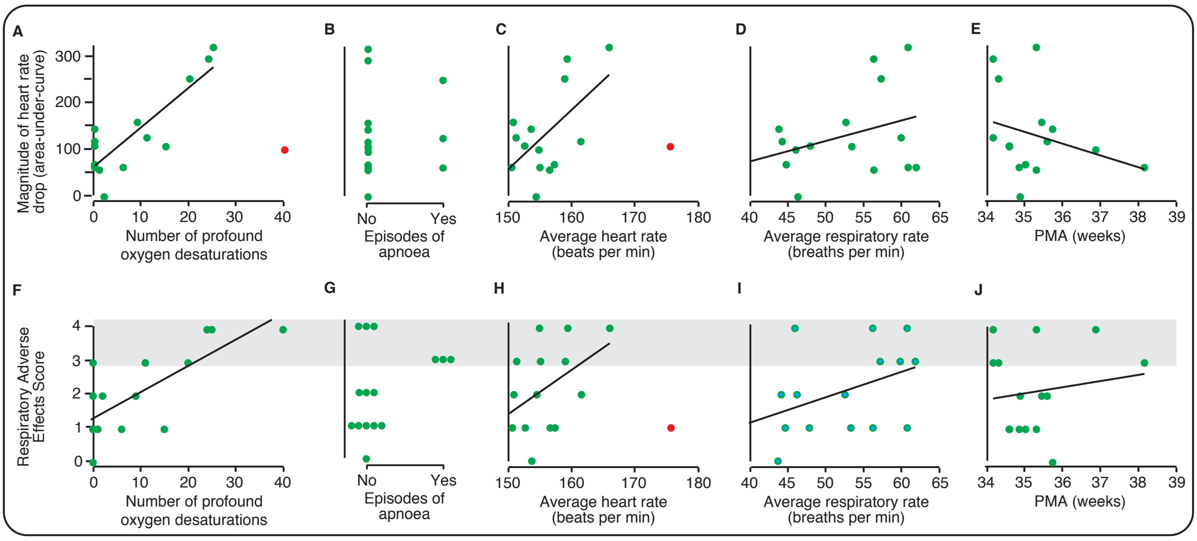

Supplementary Figure 3: Variability in baseline physiological stability correlates with the magnitude of the heart rate drop and the level of respiratory adverse effects. (A-E) The relationship between the magnitude of the heart rate drop following morphine administration and baseline variables (physiological stability and PMA). (F-J) The relationship between the Respiratory Adverse Effects Score following morphine administration and baseline variables. Black lines indicate the lines-of-best-fit from univariate linear regression, fitted excluding outliers (red dots). Grey shading indicates infants who were treated for respiratory adverse effects.

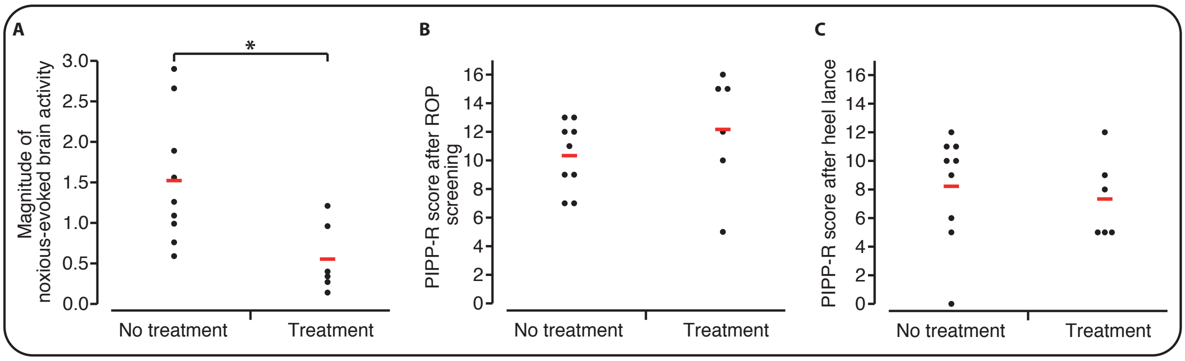

Supplementary Figure 4: Assessment of pain-related outcomes in infants who received morphine according to treatment for respiratory adverse effects. (A) Magnitude of the noxious-evoked brain activity following heel lance in the infants who received morphine and did not receive treatment for respiratory adverse effects (No treatment, N = 9 infants) and the infants who received morphine and did receive treatment for respiratory adverse effects (Treatment – NIPPV or an increase in respiratory support, N = 6 infants). (B) The PIPP-R score following ROP screening according to treatment for respiratory adverse effects. (C) The PIPP-R score following heel lance. Each infant's responses are shown as in black, horizontal red lines indicate the mean in each group, * $p<0.05$.

Supplementary Tables

Supplementary Table 1: Individual infant baseline physiological stability and post-morphine administration adverse effects

Individual values for each infant's 5 baseline predictor variables (blue, baseline physiological stability and PMA [postmenstrual age]) and the post-morphine administration adverse effect measures (green). Each row indicates a single infant.

PMA (weeks + days)	Number of profound oxygen desaturations	Infant experienced episodes of apnoea	Mean respiratory rate (breaths per minute)	Mean heart rate (beats per minute)	Magnitude of heart rate drop (area-under-the-curve)	Respiratory Adverse Effects Score	Cardiorespiratory Adverse Effects Score
34+2	20	Yes	57	159	250	3	7
38+1	0	Yes	62	155	62	3	4
36+6	40	No	46	155	100	4	6
34+1	24	No	56	159	293	4	8
35+2	25	No	61	166	317	4	8
34+6	2	No	46	154	0	2	2
35+0	0	No	45	157	68	1	2
35+4	0	No	44	162	117	2	4
35+2	1	No	56	157	57	1	2
34+6	6	No	61	151	62	1	2
34+4	0	No	48	153	108	1	3
35+5	0	No	44	154	144	0	2
35+3	9	No	53	151	158	2	4
34+1	11	Yes	60	151	125	3	5
34+4	15	No	53	176	107	1	3

Supplementary Table 2: Individual infant demographic details.

Individual demographic values for each infant. PMA – postmenstrual age.

PMA at study (weeks + days)	Gestational age at birth (weeks + days)	Sex	Mode of delivery	Birthweight (g)	Weight at study (g)	Mode of respiratory support in the baseline
34+2	27+6	Male	Caesarean-section (emergency)	1125	1928	Self-ventilating
38+1	30+0	Male	Spontaneous vaginal delivery	1320	2564	Low flow
36+6	26+2	Male	Caesarean-section (emergency)	870	2370	Vapotherm (HFT)
34+1	25+6	Female	Caesarean-section (elective)	710	1624	Vapotherm (HFT)
35+2	25+6	Male	Spontaneous vaginal delivery	810	2036	Low flow
34+6	29+0	Male	Spontaneous vaginal delivery	1520	2316	Self-ventilating
35+0	31+0	Male	Spontaneous vaginal delivery	1810	2406	Self-ventilating
35+4	27+4	Female	Spontaneous vaginal delivery	1230	2596	Self-ventilating
35+2	28+1	Male	Caesarean-section (emergency)	1120	2148	Self-ventilating
34+6	26+3	Male	Caesarean-section (emergency)	930	2266	Vapotherm (HFT)
34+4	30+4	Male	Spontaneous vaginal delivery	1510	1896	Self-ventilating
35+5	31+2	Male	Caesarean-section (elective)	950	1380	Self-ventilating
35+3	28+4	Male	Spontaneous vaginal delivery	1090	2388	Self-ventilating
34+1	30+1	Female	Caesarean-section (emergency)	1010	1460	Self-ventilating
34+4	23+5	Male	Spontaneous vaginal delivery	600	1350	Vapotherm (HFT)

Supplementary Table 3: Respiratory Adverse Effects and Heart Rate Score. Infants were assigned a Respiratory Adverse Effects Score based on episodes of apnoea, episodes of oxygen desaturation, changes in respiratory support and treatment with NIPPV (non-invasive positive pressure ventilation). Infants were assigned a Heart Rate Score based on the magnitude of their heart rate drop (h), where M is the maximum heart rate drop across all infants.

**Respiratory Adverse Effects Score**	**Infant characteristics**
0	The infant had no episodes of apnoea (following morphine administration), no increase in oxygen desaturations, and no increase in respiratory support.
1	The infant had no episodes of apnoea and no increase in respiratory support but an increase in oxygen desaturations.
2	The infant had apnoeic episodes following morphine administration but did not require an increase in respiratory support or NIPPV.
3	The infant had an apnoea requiring NIPPV or an increase in respiratory support.
4	The infant had multiple episodes of apnoea, NIPPV and an increase in respiratory support.
**Heart Rate Score**	**Magnitude of the heart rate drop (h)**
0	$h = 0$
1	$0 < h \leq \frac{M}{4}$
2	$\frac{M}{4} < h \leq \frac{M}{2}$
3	$\frac{M}{2} < h \leq \frac{3M}{4}$
4	$\frac{3M}{4} < h \leq M$

Supplementary References

1. Hartley C, Moultrie F, Hoskin A, et al. Analgesic efficacy and safety of morphine in the Procedural Pain in Premature Infants (Poppi) study: randomised placebo-controlled trial. *Lancet* 2018; **392**: 2595–605
2. Monk V, Moultrie F, Hartley C, et al. Oral morphine analgesia for preventing pain during invasive procedures in non-ventilated premature infants in hospital: the Poppi RCT. *Effic Mech Eval* 2019; **6**
3. Slater R, Hartley C, Moultrie F, et al. A blinded randomised placebo-controlled trial investigating the efficacy of morphine analgesia for procedural pain in infants: Trial protocol [version 2; peer review: 3 approved]. *Wellcome Open Res* 2017; **1**
4. Worley A, Fabrizi L, Boyd S, Slater R. Multi-modal pain measurements in infants. *J Neurosci Methods* 2012; **205**: 252–7
5. Hartley C, Duff EP, Green G, et al. Nociceptive brain activity as a measure of analgesic efficacy in infants. *Sci Transl Med* 2017; **9**: eaah6122
6. Winkler AM, Ridgway GR, Webster MA, Smith SM, Nichols TE. Permutation inference for the general linear model. *Neuroimage* 2014; **92**: 381–97
